# Revealing the transmission mechanism and spatial spillover of carbon emission reduction caused by high-speed rail opening

**DOI:** 10.1371/journal.pone.0271585

**Published:** 2022-08-19

**Authors:** Meiyu Liu, Xiaogeng Niu, Zhenxing Tian

**Affiliations:** Natural Resource Asset Capital Research Center, Hebei GEO University, Hebei, China; Gebze Teknik Universitesi, TURKEY

## Abstract

In the context of China’s commitment to peak carbon emissions by 2030 and achieve carbon neutrality by 2060, as well as its strategy to build a strong transportation country, it is of foremost importance to study the carbon emission reduction effect of the opening of high-speed rail (HSR). This paper innovatively introduces the frequency of HSR stops as an indicator of HSR operation, and uses a time-varying difference-in-difference (DID) model, a mediating effect model and a spatial DID model to assess the direct and indirect impact, transmission mechanism, and spatial spillover effects of the opening and operation of HSR on carbon emission reduction based on a panel of 279 prefecture-level cities from 2003 to 2017. We found that the opening and operation of HSR significantly reduced urban carbon emissions. The direct transmission mechanism analysis shows that the opening of HSR can reduce carbon emissions by replacing highway passenger traffic. Indirect mechanism analysis shows that the opening of HSR can reduce carbon emissions through technological effect, structural effect and opening effect. The test of spatial spillover effect shows that the opening of HSR can promote carbon emission reduction not only in node cities, but also in neighboring cities.

## Introduction

With the promotion of ecological civilization, China has incorporated the response to climate change into the medium- and long-term planning of national socio-economic development [1]. China is currently the world’s largest carbon emitter [2], accounting for 27.8% of the world’s total carbon emissions [3]. China has committed to reduce carbon emissions per unit of GDP in 2030 by 60–65% compared with the level of 2005. China is comprehensively promoting a green transformation of economic and social development and moving towards the goal of achieving a low-carbon economy. The opening of high-speed rail (HSR) and its network operation methods have not only improved the transportation structure, but also brought opportunities for green transformation of the economy and society. In recent years, China’s HSR accelerated development [4, 5]. In 2017, "four vertical and four horizontal" HSR passenger lines were completed ahead of schedule [6], and at the end of 2021, China’s HSR mileage exceeded 40,000 km, gradually forming an "eight vertical and eight horizontal" HSR operation network. As one of the clean and efficient transportation modes [7, 8], HSR is not only an alternative to traditional transportation modes in terms of carbon emission reduction, but its "convergence" effect and "space-time compression" effect can also affect ecological development.

There is abundant research on the economic and environmental effects of the HSR. In terms of the economic effects of HSR, on the one hand, some scholars believe that the HSR can reshape the spatial pattern of China’s economy [9, 10], promote technological innovation [11], improve the industrial structure [5], and narrow the urban-rural income gap [12]. On the other hand, some scholars have the opposite attitude towards the economic effects brought by HSR, arguing that the HSR brings more of a "passing effect" that does not promote economic development and may lead to slow economic development in the peripheral areas of the node centers [13]. In terms of the environmental effects of HSR [14], on the one hand, as a low-carbon and clean transportation mode, HSR can replace traditional transportation modes and directly reduce carbon emissions in the operation process [15]. On the other hand, HSR weakens the impediment of geographical distance to factor mobility, which helps accelerate the migration and reorganization of factors such as technology and knowledge between regions, thus improving the efficiency of resource utilization and enterprise productivity in cities and reducing carbon emissions [16].

Based on the above analysis, many studies have concluded that the HSR can promote carbon emission reduction. However, there are still several shortcomings in the current research: first, most of the literature only examines the impact of the exogenous shock event of HSR on carbon emission reduction, while few studies focus on the impact of the operational status of HSR on carbon emission reduction after its opening. Second, most studies do not focus on both direct and indirect transmission mechanisms of the impact of HSR opening on carbon emission reduction. Third, when researching the carbon emission reduction effect of HSR, the spatial correlation of carbon emissions has not been considered, and the spatial effect of HSR on carbon emission reduction still needs to be further explored. Based on the above points of deficiency, we expand from the following aspects. First, considering the impact of HSR opening and operation on carbon emission reduction, this paper innovatively introduces the frequency of HSR stopovers as an indicator of HSR operation. This paper empirically examines the heterogeneity, transmission mechanism and spatial spillover effect of HSR opening and operation on carbon emission reduction in cities, overcoming the shortcomings of the dummy variables used in previous studies that ignore the differences in the intensity of HSR operation. Second, This paper incorporates the direct and indirect transmission mechanisms of the impact of HSR opening on carbon emission reduction into the theoretical framework. Combining the moderating effect model and the mediating effect model, the analysis is conducted on the moderating effect of road transportation and the mediating effect of industrial structure upgrading and technological innovation. Third, focusing on the spatial perspective, the spatial Durbin model is applied to examine the spatial role characteristics of the impact of HSR on carbon emission reduction based on the spatial weight matrix of geographical distance and economic distance.

## Mechanism analysis

The flow of production factors affects regional industrial agglomeration and diffusion. China’s goal of completing a "four-longitudinal and four-horizontal" HSR network was achieved ahead of schedule in 2017. The opening of HSR will accelerate the flow of production factors, and the central node cities (municipalities, provincial capitals and sub-provincial cities) located in the "four vertical and four horizontal" network will attract more high-quality factors, which will directly or indirectly affect regional employment, wages and economic growth, and reshape the economic distribution pattern. The allocation effect of production factors in the region can inevitably affect the industrial structure and energy use structure, and thus have an impact on carbon emissions. The detailed analysis of the impact mechanism is as follows.

First, we analyze the direct transmission mechanism of carbon emission reduction by the opening of HSR.

**Fig 1:**
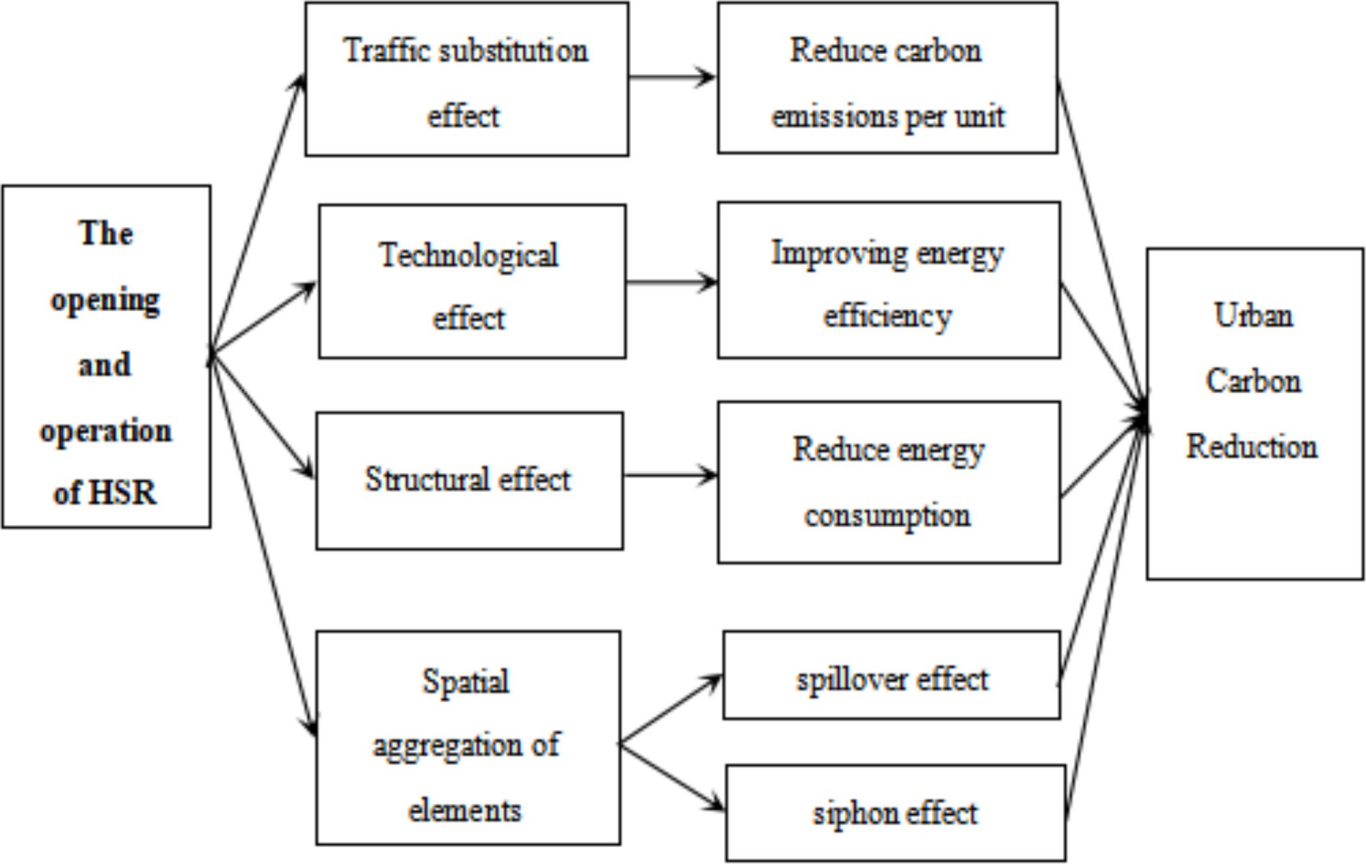
Mechanism analysis.

As one of the advanced modern transportation modes, HSR is inherently energy-saving, efficient and environmentally friendly. The opening of HSR can cause travelers to change their traditional mode. The networked operation of HSR can replace inter-city highways and ordinary rail transport [17–19], improving the regional transport structure and thus reducing carbon emissions. The substitution of either highway or rail transportation modes is accompanied by low cost and high efficiency, which does not lead to a decrease in utility, but rather promotes an increase in total social welfare. Therefore, HSR can reduce carbon emissions without reducing social welfare, which further provides useful theoretical support for the construction of HSR networks. In view of this, we put forward:

**Hypothesis 1**: The opening and operation of HSR affects urban carbon emissions by replacing highway transport (see Fig 1).

Second, we analyze the indirect transmission mechanism of carbon emission reduction by the opening of HSR.

HSR can reduce carbon emissions through the effect of technological innovation. HSR has improved inter-city accessibility, considerably shortened the spatial and temporal distances between cities [20, 21], and reduced distance barriers to technology exchange and knowledge spillover [16]. The network operation mode of HSR facilitates the technical exchange of high-end talents among enterprises, universities and research institutes in the region [22], thus promoting innovative activities and new technology development. Many studies have shown that the faster the flow of high-end talents as a carrier of knowledge and technology dependence, the greater the technology spillover effect between cities. The extension of the HSR network accelerates the diffusion of knowledge and technology to a wider spatial scale, helping to raise the level of technological innovation in nearby cities. In addition, HSR can enhance the level of human capital. Cities with HSR can attract investment from enterprises related to the HSR construction industry chain, thus expanding the market size and enhancing the attractiveness of the city to high-end talents [11]. In short, HSR can promote regional innovation and accelerate the development of green innovative technologies. Advances in green innovation technologies contribute to the achievement of carbon reduction targets.

HSR can reduce carbon emissions through structural adjustment effects. HSR belongs to the category of transportation services, and the expansion of HSR and related industries can stimulate the development of tertiary industries. The "time-space compression effect" of HSR can accelerate the circulation of production factors related to the service industry [23] and reduce the transaction costs of enterprises. It is conducive to further attracting high-end service industries to cluster in node cities and expand their radiation capacity and service radius. HSR brings production factor mobility [5] and increases local market potential. It can promote the development of tertiary industries such as catering, tourism, wholesale and retail trade, and promote the optimization and upgrading of industrial structure. In general, the tertiary sector has low energy consumption and high value-added attributes. The agglomeration of tertiary industries can facilitate the synergistic development of related manufacturing industries before and after the industrial chain, which can help improve the production efficiency of enterprises and reduce carbon emissions [24]. In view of this, we put forward:

**Hypothesis 2**: The opening and operation of HSR promotes carbon emission reduction through two effects, namely technical effect and structural effect (see Fig 1).

Third, we analyze the spatial spillover effect of the impact of HSR on carbon emissions.

Given the spatial correlation of carbon emissions, we analyze the impact of HSR on carbon emission levels and its spatial spillover effects based on a spatial perspective. On the one hand, the "time-space compression effect" of HSR shortens the distance between cities, which allows node cities to expand the scope of knowledge and technology spillover. It can improve the energy efficiency of businesses in surrounding cities and reduce carbon emissions per unit of output. On the other hand, the opening of HSR can cause low-quality production factors to cluster in neighboring cities and high-quality production factors to cluster in node cities. This "agglomeration effect" may lead to the spatial separation of production and management functions [25], i.e., the enterprise center is located in the node city, while the production department is located in the surrounding cities. In general, the "spillover" and "siphon" effects of HSR on carbon emissions of neighboring cities are as follows: First, HSR can break the market division between the node cities and the neighboring cities, so that the knowledge and technology of the node cities can serve the neighboring cities. It contributes to productivity improvements and promotes industrial integration in surrounding cities, reducing carbon emissions per unit of output. HSR can cause high-quality production factors to gather in the node cities, especially can have a "siphon effect" on neighboring cities in the early stage [26–28], and can accelerate the transfer of high-energy-consuming and high-polluting enterprises to neighboring cities, which is not conducive to carbon emission reduction in neighboring cities. HSR causes high-quality production factors to cluster in the node cities, especially have a "siphon effect" on neighboring cities in the early stage [29], and accelerate the transfer of high-energy-consuming and high-polluting enterprises, which is not conducive to carbon emission reduction in neighboring cities. In view of this, we put forward:

**Hypothesis 3**: Whether the opening and operation of HSR promotes carbon emission reduction in neighboring cities depends on the size of the "spillover effect" and "siphon effect" (see Fig 1).

## Model and data

### Model setting

The difference in difference (DID) method is one of the methods commonly used by scholars today, which effectively assesses policy effects by constructing time and individual dummy variables and multiplying the difference between the experimental and control groups. The opening of HSR provides a good "quasi-natural experiment" for the DID method. Because of the differences in the opening time of HSR, this paper adopts the time-varying DID method to evaluate the carbon emission reduction effect of HSR. The DID model is set as follows.

$${CE}_{i,t}=\alpha_{0}+\alpha_{1}{HR}_{i,t}+\gamma X_{i,t}+\mu_{i}+\delta_{t}+\varepsilon_{i,t}$$ (1)

where the subscripts i and t indicate the city and year, respectively; *CE* is carbon emissions; *HR* is the dummy variable of whether HSR is open or not, which is the core explanatory variable of this paper; *X* is a set of control variables; *μ*_*i*_ is urban fixed effects and *δ*_*t*_ is time fixed effects; *ε* is the random error term.

In order to assess the impact of the operation of HSR on urban carbon emissions after its opening, this paper innovatively introduces the frequency of HSR stops as an indicator of HSR operation. The model is set as follows.

$${CE}_{i,t}=\alpha_{0}+\alpha_{1}{HO}_{i,t}+\gamma X_{i,t}+\mu_{i}+\delta_{t}+\varepsilon_{i,t}$$ (2)

where *HO* is the variable of HSR operation. The rest of the explanatory variables are the same as in the benchmark model.

### Variable selection

#### Explained variable

Carbon emissions (*CE*). Referring to Chen et al. (2020) [30] and Sun et al. (2021) [31], this paper uses county-level carbon emission data in China from 1997 to 2017 published by CEADs, and adds county-level data to the municipal level according to the administrative divisions of China each year. From this, the total carbon emissions of 353 prefecture-level cities in China from 1997 to 2017 are summarized, and the data are logarithmically processed and combined into the panel data in this paper.

#### Explanatory variables

The opening of HSR (*HR*). First, individual dummy variables are set, with cities where HSR is open as the experimental group (*treat* = 1) and cities where HSR is not open as the control group (*treat* = 0). Second the time dummy variables are set, with *time* = 1 for the year the HSR is opened and *time* = 0 for the year the HSR is not opened. Finally, the interaction term *HR* = treat*time is set, and *HR* is a dummy variable for the city where the HSR opens, and its estimated coefficient is the impact of HSR opening on carbon emission reduction. In this paper, cities that opened HSR in the first half of the year are considered to be opened in the current year, and cities that opened HSR in the second half of the year are considered to be opened in the next year.

The operation of HSR (*HO*). The setting of the HSR opening variable can only reflect whether a city opened an HSR in that year, and it is difficult to reflect the operation status of each city after the opening of the HSR. In this paper, the frequency of HSR stops (*FRE*) is selected as an indicator of HSR operation to study the impact of HSR operation on carbon emission reduction after its opening. Multiply the frequency of HSR stops with the time dummy variable of HSR opening, i.e., *HO* = FRE*time.

#### Mediator variables

The level of innovation (*TE*). Considering that green technology innovation may have a greater impact on carbon emissions, this paper adopts the number of green invention patents granted to reflect the level of technology innovation.

The industrial structure (*IS*). The output value of the secondary industry is used to express the industrial structure variable.

#### Control variables

The level of economic development (*EC*) is expressed by the natural logarithm of GDP per capita. Population density (*POP*) is expressed by the natural logarithm of the number of people per unit area in each administrative area. Fiscal decentralization (*FS*) is expressed by the logarithm of the ratio of fiscal revenues to fiscal expenditures. The level of urbanization (*UR*) is expressed by the natural logarithm of the ratio of the built-up area to the total urban area. Investment level (*IN*) is expressed by the natural logarithm of fixed asset investment. The degree of government environmental regulation (*ER*) is expressed by the frequency of environment-related terms as a proportion of the total number of terms in provincial government work reports [32].

### Data sources

Data on the opening and operation of HSR are extracted from the National Railway Administration (NRA), 12306 China Railway website, the *China Railway Yearbook* and the *National Railway Passenger Train Timetable* for the past years. The data of carbon emissions comes from China Emission Accounts and Data-sets (CEADs). The urban green patent authorization data are from China Patent Full Text Database and China National Intellectual Property Administration (CNIPA). Except for the above variables, the data of other variables are mainly come from the *China Urban Statistical Yearbook*. After excluding cities with serious data deficiencies and administrative division changes, the data of 279 prefecture-level and above cities from 2003–2017 were finally selected as the research samples. After interpolation and supplementation of some missing data, a total of 4185 observations are obtained. Descriptive statistics of the variables are shown in Table 1.

**Table 1:** Descriptive statistics of variables.

Variable	Observation	Mean	Standard deviation	Minimum	Maximum
*CE*	4185	2.9104	0.8068	0.4248	5.4411
*HR*	4185	0.2697	0.4438	0	1
*HO*	4185	34.8322	89.2335	0	812
*TE*	4185	42.5792	194.0027	0	5073
*IS*	4185	8.2462	0.1774	7.8508	8.5436
*EC*	4185	9.8646	0.8291	3.7234	12.7859
*POP*	4185	5.7478	0.8983	1.5457	7.8824
*FS*	4185	0.8330	0.5163	-0.4353	3.664
*UR*	4185	-2.9862	1.0847	-6.7451	-0.0481
*FIN*	4185	15.4747	1.2221	12.0178	19.1611
*ER*	4185	-0.5723	0.4414	-2.3591	0.5702

## Empirical results and analysis

The empirical test of this paper is divided into four parts as follows. (1) Before the benchmark regression analysis, it is necessary to verify that the treatment and control groups satisfy the assumption of parallel trends, and then to conduct a dynamic effects analysis of the relationship between the HSR and carbon emissions. (2) Analyze the impact of the HSR on carbon emissions based on the results of the benchmark regression model. (3) Dividing the research samples by city size and geographical location to verify the heterogeneity of the impact of HSR on carbon emissions. (4) Mitigating the possible endogeneity between HSR and carbon emissions and proving the robustness of the above estimation results.

### Parallel trend test and dynamic effect analysis

**Fig 2:**
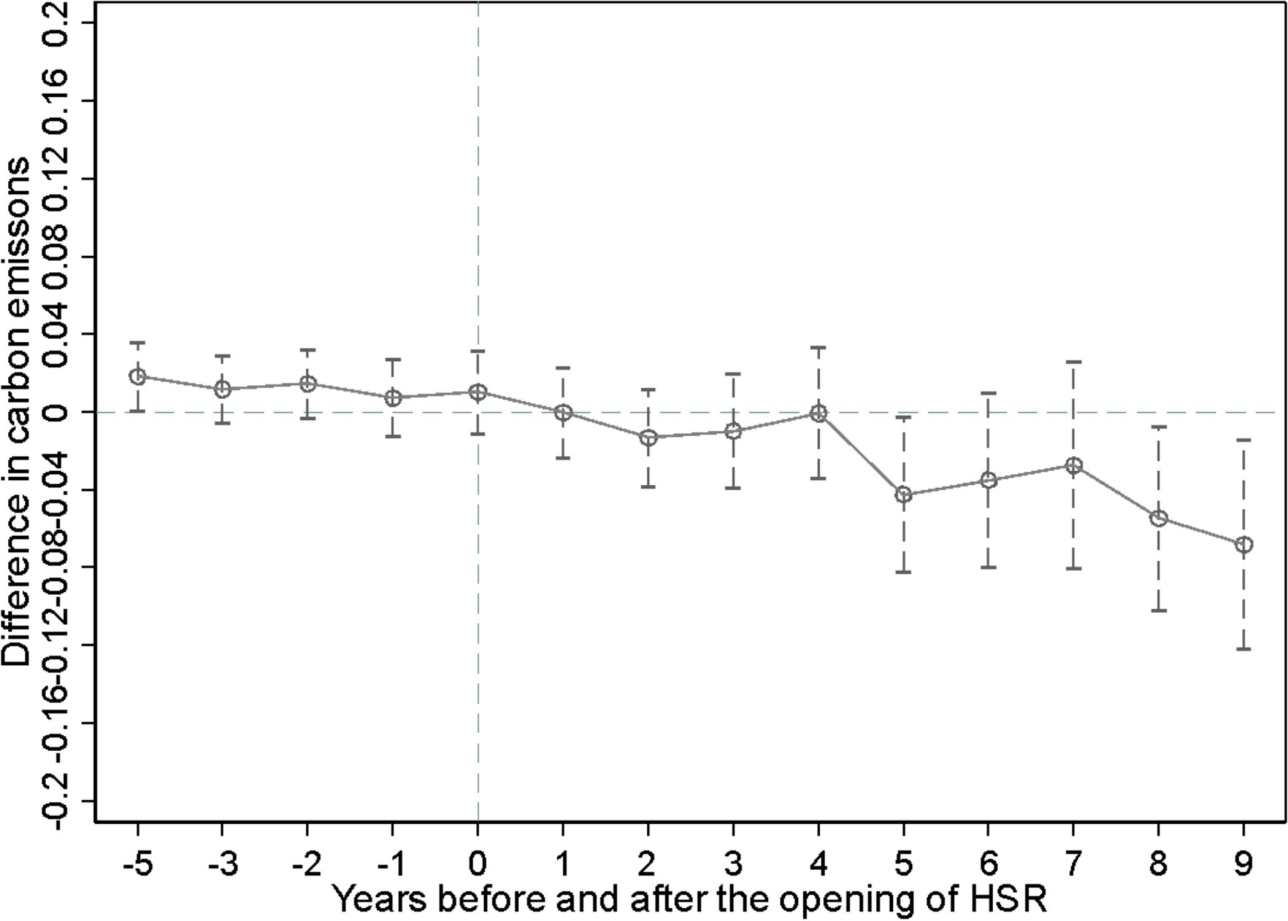
Parallel trend test and dynamic effect analysis.

The DID model needs to satisfy the parallel trend assumption before application, and referencing on Beck et al. (2010) [33], this paper incorporates the interaction term between the time dummy variable before and after the opening of the HSR and the dummy variable for the opening of the HSR into the model. Fig 2 shows the impact on carbon emissions for the first 4 years and the last 9 years of the opening of HSR. As can be seen from Fig 2, the estimated coefficient of treatment effect after the opening of HSR is negative and it starts to decrease significantly after the fourth year of HSR opening. The dynamic effect analysis indicates that there is a lag effect on the impact of HSR on carbon emissions, which may be due to the fact that it takes a certain period for the HSR to produce carbon emission reduction effects through mechanisms such as industrial structure and technological innovation.

### Analysis of benchmark model regression results

The results of the benchmark model regression are presented in Table 2. It can be seen that the coefficient of the HSR opening variable is negative with or without the inclusion of control variables (column 1 and column 2) and is significant at the 1% level, indicating that the opening of HSR can promote carbon emission reduction. To mitigate the bias caused by omitted variables, subsequent models were analyzed using a two-way fixed effects model. In terms of the economic significance of the estimated coefficients, cities with HSR are on average 2.17% lower in carbon emissions compared to cities without HSR, all else being equal. The regression results of the benchmark model in Table 2 show that the coefficient of HSR operation is significantly negative (column 3 and column4), regardless of whether the control variables are included, indicating that the carbon reduction effect is more obvious in "busy" cities with HSR.

**Table 2:** Regression results of the benchmark model.

Variable	(1)	(2)	(3)	(4)
HR	-0.0217*** (0.0052)	-0.0183*** (0.0050)		
HO			-0.0003*** (0.0000)	-0.0002*** (0.0000)
EC		0.0682*** (0.0162)		0.0583*** (0.0154)
POP		0.1496*** (0.0376)		0.1725*** (0.0377)
FS		0.0256*** (0.0088)		0.0227*** (0.0086)
UR		0.0285*** (0.0049)		0.0273*** (0.0048)
IN		0.0492*** (0.0066)		0.0422*** (0.0064)
ER		0.0551*** (0.0069)		0.0574*** (0.0068)
_Cons	3.6821*** (0.0250)	1.3556*** (0.2096)	3.7334*** (0.0250)	1.4572*** (0.2080)
Time fixed	YES	YES	YES	YES
City fixed	YES	YES	YES	YES
N	4185	4185	4185	4185
R^2^	0.9873	0.9888	0.9877	0.9890

Note

***, **, and * represent significance levels of 1%, 5%, and 10%, respectively; the values in parentheses are standard errors.

### Analysis of heterogeneity

#### Geographical location heterogeneity

There are significant differences in factor endowments and policy focus among cities in different geographical locations. This paper divides the research samples into eastern and central and western regions to examine the heterogeneity of urban carbon emissions from the opening and operation of HSR. The detailed results are presented in Table 3. It can be seen that the opening of HSR can significantly promote carbon emission reduction in the central and western regions, but increases carbon emissions in the eastern regions. The reason may be that the transportation base in the eastern region is developed and the transportation means are diversified, so it is difficult to show the unique advantages of the HSR opening, while the transportation means in the central and western regions are less selective, so the unique advantages of the HSR opening are easily highlighted. HSR operations contribute to carbon emission reduction in the eastern and central and western regions. The increase in the frequency of HSR stops can better utilize the emission reduction effect of HSR, which further provides theoretical support for the construction of the HSR network.

**Table 3:** Results of heterogeneity analysis.

Variable	Geographical location				Urban scale			
	Eastern Regions		Central and Western Regions		Large Cities		Small and Medium-sized Cities	
*HR*	0.0148*** (0.0072)		-0.0306*** (0.0065)		0.0047 (0.0048)		-0.1229*** (0.0448)	
*HO*		-0.0001*** (0.0000)		-0.0001*** (0.0000)		-0.0002*** (0.0000)		-0.0013* (0.0006)
_Cons	3.0486*** (0.1785)	3.7004*** (0.0232)	1.5679*** (0.1869)	1.5484*** (0.1869)	2.9721*** (0.1259)	3.1201*** (0.1251)	-1.2449* (0.6759)	-1.1332* (0.6813)
Variable control	YES	YES	YES	YES	YES	YES	YES	YES
City fixed	YES	YES	YES	YES	YES	YES	YES	YES
Time fixed	YES	YES	YES	YES	YES	YES	YES	YES
R^2^	0.9893	0.9780	0.9568	0.9633	0.9898	0.9782	0.9658	0.9651
N	1500	1500	2685	2685	3750	3750	435	435

Note

***, **, and * represent significance levels of 1%, 5%, and 10%, respectively; the values in parentheses are standard errors.

#### Urban scale heterogeneity

The results of the above theoretical analysis show that the opening of HSR can significantly promote carbon emission reduction, and the opening of HSR shows significant differences in economic development and environmental pollution in cities of different sizes. The results of the above theoretical analysis show that the opening of HSR can significantly contribute to carbon emission reduction, and it shows differences in cities of different sizes. On the one hand, the "siphon effect" of large cities can promote the integration of factor resources, thus improving the efficiency of resource utilization. On the other hand, the "urban diseases" such as population expansion, traffic congestion and environmental degradation in large cities are becoming increasingly serious, and carbon emission reduction is facing difficulties. To analyze the differences in the impact of HSR opening on different city sizes, this paper divides the city sizes into large cities and small and medium-sized cities. The results by city size are reported in Table 3. It can be seen that the opening of HSR can significantly promote carbon emission reduction in small and medium-sized cities, but it is difficult to suppress carbon emission in large cities, and even the impact on carbon emission in large cities is positive. This indicates that the emission reduction effect of the opening of HSR in small and medium-sized cities is better than that of large cities. The reason may be that the opening of HSR has a small marginal impetus on the transmission mechanisms of industrial structure and technological innovation in large cities, making it difficult to stimulate changes in the economic structure within cities. However, for some small and medium-sized cities, the opening of HSR has a stronger marginal impetus on their internal economic structural transformation, which is more helpful to promote carbon emission reduction in small and medium-sized cities through the transmission mechanism.

### Endogeneity problem and robustness tests

#### Endogeneity problem

Although the DID method can effectively alleviate the endogeneity problem caused by individual heterogeneity, it is undeniable that the location of HSR stations is not random, and the planners’ preference for economy and location influence the location of HSR stations. If these unobservable factors affect both HSR opening and urban carbon emissions, it will lead to endogeneity of the explanatory variable "*HR*" and thus bias the estimation results. In this paper, the instrumental variable for the opening of HSR is constructed in terms of the topographic relief of each city. A reasonable explanation for this approach is as follows. On the one hand, topographic undulation as an exogenous geographic variable is generally uncorrelated with economic and social variables and satisfies the endogeneity assumption. On the other hand, the topographic undulation reflects the difficulty of building HSR, that is, the more undulating the topography of the city, the more difficult it is to build HSR, satisfying the correlation assumption. Since the topographic undulation of each city does not vary with time, this paper constructs the interaction term of topographic undulation and time dummy variables as instrumental variables for the opening of HSR. The estimation results of the instrumental variable model are presented in Table 4.

**Table 4:** Endogeneity problem.

**Variable**	**Regression Results of the First Stage**		
	Explained Variable: *HR*		
TOR*2003	0.0926*** (0.0244)	TOR*2011	-0.0723*** (0.0235)
TOR*2004	0.0550** (0.0242)	TOR*2012	-0.0714*** (0.0235)
TOR*2005	0.0207 (0.0238)	TOR*2013	-0.0661*** (0.0236)
TOR*2006	-0.0029 (0.0237)	TOR*2014	0.0594** (0.0237)
TOR*2007	-0.0359 (0.0236)	TOR*2015	0.0666*** (0.0239)
TOR*2008	0.0174 (0.0238)	TOR*2016	0.1031*** (0.0240)
TOR*2009	-0.0228 (0.0234)	TOR*2017	0.1083*** (0.0249)
TOR*2010	-0.0555** (0.0235)	Variable control	YES
**Variable**	**Regression Results of the Second Stage**		
	Explained Variable: *CE*		
*HR*	-0.7445*** (0.1310)	*EC*	0.0353*** (0.0118)
*POP*	-0.0910*** (0.0148)	*UR*	0.0327*** (0.0082)
*FS*	-0.2787 (0.0211)	*ER*	0.0574*** (0.0070)
*IN*	0.6071*** (0.0236)	R^2^	0.6265
Time fixed	YES	City fixed	YES
N	4185	F-statistic of the first stage	71.46

Note

***, **, and * represent significance levels of 1%, 5%, and 10%, respectively; the values in parentheses are standard errors.

In the first-stage regression results, the coefficient of the interaction term between topographic undulation and the year dummy variable is overwhelmingly significantly negative, indicating that the higher the topographic undulation of the city, the more difficult it is to build HSR. The F-statistic is greater than 10, which rejects the original hypothesis of weak instrumental variables and indicates that the instrumental variables are appropriately selected. From the second stage regression results, it is clear that the opening of HSR can significantly reduce carbon emissions, which is consistent with the findings of the previous research. This indicates that the core conclusion of "the opening of HSR can promote carbon emission reduction" is credible.

#### Robustness tests

In this paper, the above regression results are tested for robustness in the following ways. (1) Using the PSM-DID method. Since the construction of HSR stations is inevitably influenced by the location and economic development level, this paper conducts regression based on the propensity score matching (PSM) combined with DID method. The experimental and control groups were first divided according to the propensity score values, and then estimated using the DID method. The results are presented in column (1) of Table 5. It can be seen that the coefficient of HSR opening is significantly negative. (2) Lagging all explanatory variables by one period. In this paper, we lag the core explanatory variables with all control variables by one period to mitigate endogeneity. The results are presented in column (2) of Table 5. The significant negative coefficient for the opening of HSR further supports the robustness of the study findings. (3) Excluding the special samples. Due to their high administrative level, provincial capitals and municipalities directly under the central government may be influenced by a series of unobservable factors such as planners’ economic and locational preferences, which can lead to biased estimation results. Therefore, these cities are excluded from the regressions in this paper and the results are reported in column (3) of Table 5. It can be seen that the coefficient for the opening of the HSR is still significantly negative, proving the robustness of the above findings.

**Table 5:** Robustness tests.

Variable	(1)	(2)	(3)
	PSM-DID	Lagging all explanatory variables by one period	Excluding the special samples
*HR*	-0.0172*** (0.0052)	-0.0224*** (0.0053)	-0.0164*** (0.0052)
_Cons	2.3673*** (0.4369)	0.0463 (0.2535)	1.4733*** (0.2096)
Variable control	YES	YES	YES
Time fixed	YES	YES	YES
City fixed	YES	YES	YES
N	3975	3906	3675
R^2^	0.9849	0.9863	0.9840

Note

***, **, and * represent significance levels of 1%, 5%, and 10%, respectively; the values in parentheses are standard errors.

## Analysis of the transmission mechanism

### Direct transmission mechanism

From the above benchmark model results, it is clear that the opening and operation of HSR has a significant carbon emission reduction effect. Based on hypothesis 1, this paper assumes that HSR may directly affect urban carbon emissions by substituting highway passenger traffic. To verify whether this effect exists, this paper refers to the research of Li and Luo (2020) to construct a regression equation for the effect of the linkage effect between HSR and highway passenger transportation on carbon emissions [34]. The specific steps are as follows. (1) Constructing a model to test the impact of HSR on highway passenger traffic. (2) Incorporating the interaction term of HSR and highway passenger traffic into the model. (3) Determining the linkage effect of HSR and highway passenger transportation on carbon emissions based on the interaction term coefficient. The models are set as follows.

$${HW}_{i,t}=\beta_{0}+\beta_{1}{HR}_{i,t}+{\varphi X}_{i,t}+\mu_{i}+\delta_{t}+\varepsilon_{i,t}$$ (3)

$${HW}_{i,t}=\omega_{0}+\omega_{1}{HO}_{i,t}+{\theta X}_{i,t}+\mu_{i}+\delta_{t}+\varepsilon_{i,t}$$ (4)

$${CE}_{i,t}=\tau_{0}+\tau_{1}{HR}_{i,t}+\tau_{2}{HR}_{i,t}*{HW}_{i,t}+\vartheta X_{i,t}+\mu_{i}+\delta_{t}+\varepsilon_{i,t}$$ (5)

$${CE}_{i,t}=\rho_{0}+\rho_{1}{HO}_{i,t}+\rho_{2}{HO}_{i,t}*{HW}_{i,t}+\sigma X_{i,t}+\mu_{i}+\delta_{t}+\varepsilon_{i,t}$$ (6)

where *HW* is the highway passenger traffic and other variables are interpreted in the same way as Eq (1). *τ*_2_ and *ρ*_2_ are the core estimation parameter, which indicate the carbon reduction effect of linking HSR and highway passenger transport. If *τ*_2_ and *ρ*_2_ are less than zero, it means that the carbon reduction effect of HSR is more obvious in cities with a higher volume of highway passenger traffic. The regression results are presented in Table 6.

**Table 6:** Direct transmission mechanism.

Variable	Explained Variable: *HW*				Explained Variable: *CE*			
	(1)	(2)	(3)	(4)	(5)	(6)	(7)	(8)
*HR*	-0.0408*** (0.0115)	-0.0414*** (0.0117)						
*HO*			-0.0002*** (0.0000)	-0.0003*** (0.0000)				
HR*HW					-0.0242*** (0.0057)	-0.0266*** (0.0054)		
HO*HW							-0.00003*** (0.0000)	-0.00003*** (0.0000)
_Cons	0.8334*** (0.00451)	0.8297** (0.3848)	0.7647*** (0.0458)	0.6915* (0.3838)	3.6807*** (0.0250)	1.3574*** (0.2094)	3.7240*** (0.0251)	1.4498*** (0.2086)
Variable control	NO	YES	NO	YES	NO	YES	NO	YES
Time fixed	YES	YES	YES	YES	YES	YES	YES	YES
City fixed	YES	YES	YES	YES	YES	YES	YES	YES
N	4185	4185	4185	4185	4185	4185	4185	4185
R^2^	0.2308	0.2941	0.2258	0.2919	0.4466	0.9861	0.4942	0.9858

Note

***, **, and * represent significance levels of 1%, 5%, and 10%, respectively; the values in parentheses are standard errors.

In columns (1), (2), (3), and (4), the coefficients of HSR are negative and significant at the 1% level regardless of the inclusion of control variables, proving that there is a significant substitution effect of HSR opening and operation on highway passenger transportation. The coefficients of the interaction terms for HSR and high passenger transport in columns (5), (6), (7) and (8) are all negative and significant at the 1% level. Not only does it show that HSR will reduce carbon emissions by substituting highway passenger traffic, but the more significant the carbon reduction effect of HSR is in cities with a higher volume of highway passenger traffic. Hypothesis 1 is verified.

### Indirect transmission mechanism

From the analysis of the theoretical mechanism, it is clear that the opening of HSR may affect carbon emission reduction through two transmission mechanisms such as technological effect and structural effect. To verify hypothesis 2, this paper uses a mediating effects model to identify the two transmission mechanisms. The mediating effect models constructed in this paper are as follows.

$${CE}_{i,t}=\varphi_{0}+\varphi_{1}{HR}_{i,t}+\theta X_{i,t}+\mu_{i}+\delta_{t}+\varepsilon_{i,t}$$ (7)

$$M_{i,t}=\pi_{0}+\pi_{1}{HR}_{i,t}+\theta^{\prime }X_{i,t}+\mu_{i}+\delta_{t}+\varepsilon_{i,t}$$ (8)

$${CE}_{i,t}=\tau_{0}+\tau_{1}{HR}_{i,t}+\tau_{2}M_{i,t}+\theta^{\prime \prime }X_{i,t}+\mu_{i}+\delta_{t}+\varepsilon_{i,t}$$ (9)

where *M* is the mediating variable and the rest of the variables are explained the same as in the benchmark model (1). According to the principle of mediating effect, if the coefficients *φ*_1_, *π*_1_ and *τ*_1_ of HSR opening in the three models are significant and the absolute value of the coefficient of *τ*_1_ becomes smaller or the significance level becomes lower compared to *φ*_1_, then it proves that there is a mediating effect. The mediating effect model regression results are presented in Table 7.

**Table 7:** Indirect transmission mechanism.

Mediating effects	Technological effect			Structural effect		
	(1)	(2)	(3)	(4)	(5)	(6)
Variable	CE	TL	CE	CE	AIS	CE
*HR*	-0.0183*** (0.0053)	45.4683*** (5.1962)	-0.0131*** (0.0053)	-0.0183*** (0.0053)	-0.0851*** (0.0238)	-0.0156*** (0.0050)
M			-0.0001*** (0.00002)			0.0284*** (0.0059)
_Cons	1.3556*** (0.2096)	2343.3780*** (259.3176)	1.5757*** (0.2094)	1.3556*** (0.2096)	5.5346*** (0.1676)	0.4513* (0.0050)
Variable control	YES	YES	YES	YES	YES	YES
Time fixed	YES	YES	YES	YES	YES	YES
City fixed	YES	YES	YES	YES	YES	YES
N	4185	4185	4185	4185	4185	4185
R^2^	0.9854	0.5975	0.9855	0.9854	0.7823	0.9855

Note

***, **, and * represent significance levels of 1%, 5%, and 10%, respectively; the values in parentheses are standard errors.

In columns (1) and (4), the coefficient of HSR on carbon emissions is negative and significant at the 1% level, indicating that the opening of HSR promotes carbon emission reduction, which is consistent with the previous findings. As shown in columns (2) and (5), the coefficients of the opening of HSR on technological innovation and industrial structure are negative and significant at the 1% level, indicating that the opening of HSR can expand the technological and structural effects of the city. The absolute values of the estimated coefficients of the opening of HSR in columns (3) and (6) are smaller than those in columns (1) and (4), respectively. Hypothesis 2 is verified.

## Analysis of spatial spillover effects

The previous analysis shows that HSR significantly reduces carbon emissions. If spatial effects are considered, what is the impact of HSR on carbon emission reduction in surrounding cities? Does it promote carbon reduction in neighboring cities or inhibit it? Based on the above problems, this paper uses a spatial difference-in-difference (SDID) model for validation. The traditional DID model strictly assumes that individuals are independent of each other. However, according to theoretical analysis, the "time-space compression" effect of HSR opening generates positive knowledge and technology spillover to neighboring cities, which can promote carbon emission reduction in neighboring cities. To further analyze the spatially differentiated effects of HSR on carbon emissions, this paper uses the SDID model to research. The models are set as follows.


$${CE}_{i,t}=\alpha_{l}I_{n}+\gamma \sum W_{i,j}{CE}_{i,t}+\alpha_{1}{HR}_{i,t}+X_{i,t}\beta +\theta \sum W_{i,j}(\alpha_{1}{HR}_{i,t})+\mu_{i}+\delta_{t}+\varepsilon_{i,t}$$ (10)

$${CE}_{i,t}=\alpha_{l}I_{n}+\gamma \sum W_{i,j}{CE}_{i,t}+\alpha_{1}{HO}_{i,t}+X_{i,t}\beta +\theta \sum W_{i,j}(\alpha_{1}{HO}_{i,t})+\mu_{i}+\delta_{t}+\varepsilon_{i,t}$$ (11)

where *γ* and *θ* represent the coefficients of the spatial lagged terms of the explanatory and explanatory variables, respectively. *α*_*l*_ is the constant term, *I*_*n*_ is the unit matrix, and 3 is the spatial weight matrix. In this paper, two types of spatial weight matrix are used. The first category is the spatial weight matrix of geographic distances, where the element *W*_*ij*_ in the matrix represents the inverse geographic distance between city *i* and city *j*. Considering the association of economic activities between cities, the second category uses a spatial weight matrix of economic distances, and the element *W*_*ij*_ in the matrix represents the inverse of the difference in absolute values of annual average GDP between city *i* and city *j*.

The spatial correlation of carbon emissions needs to be validated before model estimation. The global Moran’s *I* can examine the spatial correlation of carbon emissions. In this paper, we measure the global Moran’s *I* under the two spatial weight matrixes. Detailed results are presented in Table 8. It can be seen that Moran’s *I* is significantly positive for all years, both under the W_1_ weight matrix and the W_2_ weight matrix. This indicates a clear spatial correlation of carbon emissions.

**Table 8:** Moran’s I under W_1_ and W_2_ weight matrixes.

**Matrix**	**Year**	**2003**	**2004**	**2005**	**2006**	**2007**	**2008**	**2009**	**2010**
W_1_	Moran’s *I*	0.107*** (14.109)	0.111*** (15.234)	0.118*** (16.238)	0.118*** (16.218)	0.121*** (16.589)	0.121*** (16.656)	0.118*** (16.275)	0.117*** (16.132)
	Year	2011	2012	2013	2014	2015	2016	2017	
	Moran’s *I*	0.112*** (15.362)	0.112*** (15.397)	0.104*** (14.335)	0.103*** (14.195)	0.106*** (14.649)	0.106*** (14.623)	0.098*** (13.587)	
**Matrix**	**Year**	**2003**	**2004**	**2005**	**2006**	**2007**	**2008**	**2009**	**2010**
W_2_	Moran’s *I*	0.101*** (3.691)	0.106*** (3.859)	0.112*** (4.067)	0.116*** (4.207)	0.119*** (4.302)	0.122*** (4.398)	0.121*** (4.380)	0.123*** (4.439)
	Year	2011	2012	2013	2014	2015	2016	2017	
	Moran’s *I*	0.128*** (4.631)	0.125*** (4.528)	0.120*** (4.335)	0.116*** (4.190)	0.114*** (4.126)	0.113*** (4.093)	0.114*** (4.145)	

Note

***, **, and * represent significance levels of 1%, 5%, and 10%, respectively; the values in parentheses are Z-statistics.

The estimation results of the SDID model based on the Maximum Likelihood Estimate (MLE) are presented in Table 9. Columns (1) and (2) represent the effect of HSR on carbon emissions under the inverse geographic distance spatial weight matrix, respectively. It can be seen that the coefficients of both HSR opening and HSR operation are negative, but the coefficient of HSR opening is not significant, which indicates that the increased frequency of HSR stops has a more significant carbon emission reduction effect on local compared to HSR opening. The spatial lag coefficients of both HSR opening and HSR operation are significantly negative, indicating that both HSR opening and increased frequency of HSR stops can effectively promote carbon emission reduction in surrounding cities.

**Table 9:** SDID model estimation results.

Variable	Matrix: W_1_		Matrix: W_2_	
	(1)	(2)	(3)	(4)
*HR*	-0.0028 (0.0045)		-0.0102** (0.0049)	
W*HR	-0.0957*** (0.0136)		-0.0331*** (0.0104)	
*HO*		-0.0001*** (0.0000)		-0.0001*** (0.0000)
W*HO		-0.0762*** (0.0134)		-0.0173** (0.0105)
_Cons	-4.8751*** (0.7433)	-4.8092*** (0.7283)	-3.5246*** (0.4875)	-3.6951*** (0.4921)
Variable control	YES	YES	YES	YES
Time fixed	YES	YES	YES	YES
City fixed	YES	YES	YES	YES
N	4464	4464	4464	4464
R^2^	0.8937	0.8997	0.8835	0.8855

Note

***, **, and * represent significance levels of 1%, 5%, and 10%, respectively; the values in parentheses are standard errors.

Columns (3) and (4) represent the effect of HSR on carbon emissions under the economic distance spatial weight matrix, respectively. It can be seen that the coefficients of both HSR opening and HSR operation are significantly negative, indicating that both of them can significantly contribute to local carbon emission reduction. The coefficient of the spatial lag term of HSR opening and HSR operation is also significantly negative, indicating that HSR opening and higher frequency of HSR stops effectively promote carbon emission reduction in cities with similar levels of economic development.

## Conclusions and discussion

This paper analyzes the impact of HSR on carbon emissions and its transmission mechanism and spatial spillover effect using data from 279 prefecture-level cities from 2003 to 2017. The main conclusions of the research are as follows. (1) Overall, the opening of HSR has a significant carbon reduction effect, and the conclusion remains reliable after mitigating endogeneity and after a series of robustness tests. (2) The transmission mechanism analysis represents that the opening of HSR reduces carbon emissions by substituting highway passenger traffic, and promotes urban carbon emission reduction through a series of transmission mechanisms such as technological effect and structural effect. (3) In terms of urban scale, the emission reduction effect of HSR opening is more significant for small and medium-sized cities, and HSR operation has a significant emission reduction effect for all scales. In terms of geographical location, the opening of HSR has a more significant carbon reduction effect on the central and western regions. (4) The analysis of spatial spillover effects represents that the opening and operation of HSR promote carbon emission reduction in the surrounding areas.

This paper confirms that HSR has carbon emission reduction effects based on local and neighboring locations perspectives. This conclusion not only enriches the theory of HSR economics, but also has important practical significance for the sustainable development of China’s economy. The following implication can be drawn from the findings of this paper: (1) To establish a modern HSR operation pattern in China and to realize the autonomy and intelligence of HSR construction. (2) To accelerate the restructuring of transport and improve the efficiency of transport organization. Through various measures to promote the market share of HSR in intercity passenger transport, strengthen the interconnection and complementary advantages of HSR and road transport. (3) Completing the green technology innovation system and innovation results transformation chain. Taking the green transformation and upgrading of industry as the guide, actively promoting the development of tertiary industry. (4) The opening of HSR is appropriate to give more consideration to small and medium-sized cities and central and western regions to promote balanced economic development. (5) Establishment of cross-regional joint carbon reduction mechanism to coordinate the process of energy saving and carbon reduction.

## Supporting information

S1 Code (DO)

S1 Data (DTA)
